# Suppressive role of myeloid-derived suppressor cells (MDSCs) in the microenvironment of breast cancer and targeted immunotherapies

**DOI:** 10.18632/oncotarget.11352

**Published:** 2016-08-17

**Authors:** Dawei Shou, Liang Wen, Zhenya Song, Jian Yin, Qiming Sun, Weihua Gong

**Affiliations:** ^1^ Department of Surgery, Second Affiliated Hospital of School of Medicine, Zhejiang University, Hangzhou City, People's Republic of China; ^2^ Department of Comprehensive Medicine, Second Affiliated Hospital of School of Medicine, Zhejiang University, Hangzhou City, People's Republic of China; ^3^ Department of Breast Surgery, Tianjin Medical University Cancer Institute and Hospital, National Clinical Research Center for Cancer, City Key Laboratory of Tianjin Cancer Center, Tianjin, People's Republic of China; ^4^ Department of Biochemistry, School of Medicine, Zhejiang University, Hangzhou City, People's Republic of China

**Keywords:** myeloid-derived suppressor cells, breast cancer, microenvironment, immunotherapy

## Abstract

Myeloid-derived suppressor cells (MDSCs) play a pivotal role in promoting tumor growth and metastasis and can even decrease the efficacy of immunotherapy. In breast cancer, MDSCs are recruited mainly by breast cancer cells to form a tumor-favoring microenvironment to suppress the anti-tumor immune response. In addition, MDSCs can react directly with breast cancer cells. In this paper, we describe several ways to recruit MDSCs in breast cancer, including breast cancer cell-derived cytokines and chemokines. The intracellular pathways in MDSCs during recruitment are classified as the STAT3-NF-κB-IDO pathway, the STAT3/IRF-8 pathway and the PTEN/Akt pathway. MDSCs act on T cells and NK cells to suppress the body's immunity, and via IL-6 trans-signaling, promote breast cancer directly. We further describe MDSC-targeted immune therapies for breast cancer, which are classified as: preventing the formation of MDSCs, eliminating MDSDCs, and reducing the products of MDSCs. Furthermore, MDSC-targeted immunotherapy potentiates the effect of the other immunotherapies. Based on the facts that MSDCs have significant roles in breast cancer malignant behaviors and can be suppressed by various strategies, we do believe MDSC-targeted immunotherapy presents a broad prospect in the future.

## INTRODUCTION

Breast cancer is one of the major types of cancer in women, accounting for many cancer related deaths in women [1]. There are three important markers for breast cancer: the estrogen receptor (ER), the progesterone receptor (PR), and Human epidermal growth factor receptor (HER2/neu). Based on the expression of ER, PR, HER2, breast cancer can be classified into four types: Luminal A, Luminal B, HER2(+) and triple negative breast cancer. Treatments for breast cancer are mainly based on this classification, including surgery, chemotherapy, radiotherapy, endocrine treatment and targeted therapy [2]. However, this classification based therapy does not take the complicated tumor immune situation into consideration, which has been shown to influence the curative effect.

Myeloid-derived suppressor cells (MDSCs) are a heterogeneous population of myeloid progenitor cells, comprising immature granulocytes, macrophages, and dendritic cells (DCs) [3]. In mice, MDSCs express both the myeloid lineage differentiation antigen Gr1 and CD11b [4, 5]. Human MDSCs are less defined but always characterized as Lin^−/Lo^CD33^+^CD11b^+^HLA-DR^−^ cells, which are further classified into CD14^+^ monocytic MDSCs (Mo-MDSCs) and CD15^+^ granulocytic MDSCs (G-MDSCs) [3, 6, 7]. In addition, other specific types of MDSCs have been defined recently, such as CD45^+^CD33^+^CD13^+^CD14^−^CD15^−^ cells [8] and CD11b^low^CD16^−^ cells [9]. MDSCs are recruited to inhibit the innate and adaptive immune responses by suppressing CD4^+^ T cells, CD8^+^ T cells and NK cells in conditions such as inflammation, autoimmune disease and cancer progression [10–12].

In recent years, many studies on breast cancer and MDSCs have been performed. Breast cancer cells can recruit tumor-infiltrating leukocytes, including regulatory T cells (Treg), MDSCs and type 2 macrophages, to form a tumor-promoting microenvironment, which downregulates the anti-tumor immunity [13]. Conversely, MDSC-induced immune tolerance facilitates the progression and metastasis of breast cancer. Clinically, circulating MDSC levels have been shown to correlate with breast cancer stage and metastatic tumor burden [7]. In addition, higher MDSC frequencies might correlate with increased rate of recurrence and metastasis of breast cancer [9, 14, 15]. By contrast, patients with lower levels of circulating MDSCs have a higher possibility of achieving a pathological complete response (pCR) [16]. This study reviewed current research on MDSCs and breast cancer focusing on the recruitment of MDSCs, MDSCs' functions and MDSC-targeted immunotherapy.

## THE FORMATION AND RECRUITMENT OF MDSCS IN BREAST CANCER

Breast cancer cells can induce the accumulation of MDSCs from bone marrow to a local tumor site in several ways, including cancer-derived cytokines and chemokines. In breast cancer, the cytokines concerned with the differentiation of MDSCs from bone marrow progenitors include G-CSF [17], M-CSF [18], GM-CSF [19], IL-6 [20], IL-1β [21], macrophage migration inhibitory factor (MIF) [22], TGF-β1 [23]. G-CSF, M-CSF, IL-6, MIF and TGF-β1 were verified to promote the differentiation of myeloid cells into MDSCs in 4T1 mammary tumor BALB/c mice [17, 18, 20, 22, 23]. GM-CSF was proved to induce MDSCs in mice mammary cancer (MMC) FVB and FVBN202 transgenic mice [19]. All these cytokines are related to MDSC formation and expansion in breast cancer; however, the detailed role of each cytokine and their interactions in functions on MDSCs remain obscure. Interestingly, IL-1β has a bimodal effect on MDSC migration. It can augment the recruitment of MDSCs at a suitable concentration. However, when the concentration of IL-1β is too low or too high, it will reduce the recruitment of MDSCs [21]. The chemokines that participate in the migration of MDSCs in breast cancer include CXCL5 [24], CCL1 [21] and CCL2 (also known as monocyte chemotactic protein-1 (MCP-1)) [25] and CCL5 [26]. CXCL5 was reported to interact with CXCR2, which plays a pivotal role in MDSC recruitment in 4T1 BALB/c mice [24]. CCL5 cooperates with GM-CSF and G-CSF to affect the expansion of MDSCs in 4T1 BALB/c mice [26]. CCL1 and CCL2 also promote the accumulation of MDSCs and the recruitment of MDSCs in 4T1 BALB/c mice [21, 25].

**Figure 1:**
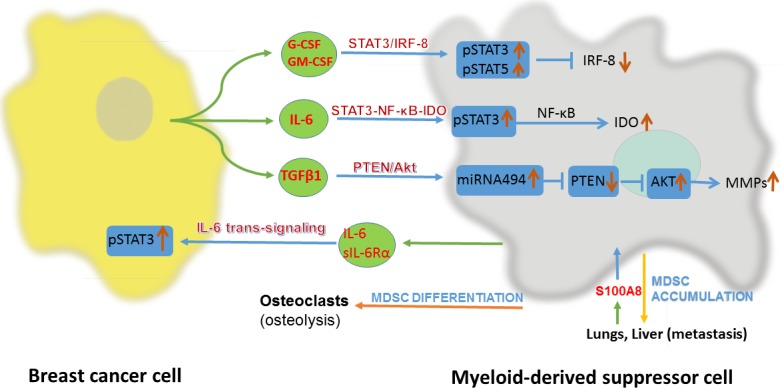
Interaction between breast cancer cell and myeloid-derived suppressor cell(MDSC) IRF-8, interferon regulatory factor-8; IDO, indoleamine 2,3-dioxygenase; PTEN, phosphatase and tensin homolog; sIL-6Rα, soluble IL-6 receptor α.

Breast cancer modulates the production of MDSC-prone cytokines and chemokines through several pathways. A study revealed that the endoplasmic reticulum disulfide oxidase ERO1-α in breast cancer can stimulate the transformation of G-CSF and CXCL1/2 from their immature forms to the mature forms posttranscriptionally rather than at the level of gene expression [27]. One study revealed that the Kruppel-like factor KLF4, a transcription factor, could induce GM-CSF production *via* CXCL5 to modulate the maintenance of MDSCs in breast cancer [24]. In addition to breast cancer cell-derived factors, there are other factors that participate in recruitment of MDSCs. One randomized clinical study indicated that psychological stress could alter the level of MDSCs in breast cancer cells *via* the upregulation of IL-1Rα, IP 10, G-CSF, and IL-6 [28]. In breast cancer, lungs and livers could highly express S100A8 and facilitates the recruitment of MDSCs in these metastatic foci, which promote the metastasis of breast cancer (Figure 1) [29].

## THE INTRACELLULAR PATHWAYS IN MDSCS IN BREAST CANCER

In breast cancer, after differentiation and recruitment, MDSCs exert their functions *via* several pathways. Firstly, it is the STAT3-NF-κB-IDO pathway. IL-6 derived from breast cancer cells can activate STAT3 in MDSCs in 4T1 BALB/c mice, which stimulates the noncanonical NF-κB subunit p52 and causes RelB translocation into the nucleus, where they interact directly with the indoleamine 2,3-dioxygenase (IDO) promoter sequence to promote expression of IDO [30]. Secondly, it is the STAT3/IRF-8 pathway. G-CSF and GM-CSF can downregulate interferon regulatory factor-8 (IRF-8) transcription *via* a STAT3- and STAT5-dependent pathway in MDSCs in 4T1 BALB/c mice. IRF-8 is a negative regulator of human MDSCs, as the downregulation of IRF-8 is accompanied by increased numbers of MDSCs [14]. Last but not least, the PTEN/Akt pathway. TGF-β1 induces the upregulation of microRNA-494 in MDSCs in 4T1 BALB/c mice, which then downregulates the expression of phosphatase and tensin homolog (PTEN). Downregulation of PTEN results in the activation of the Akt pathway (including the mTOR and NF-κB pathways) and finally increasing the expression of metalloproteinases (MMPs, including MMP2, MMP13 and MMP14), which can promote breast cancer invasion and metastasis [23]. The detailed intracellular pathways have been summarized (Figure 1).

## MDSCS PROMOTE BREAST CANCER IN VARIOUS WAYS

MDSCs have a multi-functional role in breast cancer. Except for their canonical suppression role on T cells, MDSCs also affect breast cancer cells directly and function as osteoclast progenitors to exacerbate breast cancer related osteolysis. In the breast cancer microenvironment, MDSCs suppress T cells in a variety of ways, including the arginase, ROS, RNS and NO pathways. MDSCs can produce free radical peroxynitrite (PNT) ONOO^−^ *via* ROS and RNS. PNT leads to nitration/nitrosylation of TCRs and CD8 molecules on the surface of T cells, which induce T cell tolerance [31]. MDSCs can inhibit CD3/CD28-induced T cell proliferation through a contact-dependent pathway [32]. On the other hand, MDSCs have direct effects on breast cancer cells. After activated by IL-6 derived from metastasizing breast cancer cells, MDSCs can express both IL-6 and soluble IL-6Rα, which stimulate STAT3 phosphorylation through IL-6 trans-signaling in breast cancer cells. It may contribute to invasiveness and metastasis of breast cancer [20, 33]. What's more, osteolysis, as a complication of breast cancer, is related with MDSCs. Through nitric oxide signaling and cross talk with breast cancer cells, MDSCs can differentiate into osteoclasts in the bone microenvironment to exacerbate osteolysis in metastasizing breast cancer (Figure 1) [18, 34].

## MDSC-TARGETED IMMUNOTHERAPIES

As one of the newly developed immunotherapies, there are many strategies to target MDSCs in breast cancer, such as preventing the formation of MDSCs, eliminating MDSDCs and reducing the products of MDSCs.

Firstly, prevention of MDSCs formation and accumulation can be workable. Once the production of MDSC-prone cytokines and chemokines are blocked, the formation of MDSCs decreases. Curcumin, an IL-6 inhibitor, can reduce the production of IL-6 in breast cancer to reduce the number of MDSCs [35]. BMP4, a member of TGFβ growth factor family, can reduce the expression of G-CSF in human and mice breast cancers by inhibiting NF-κB activity [17]. r84, an anti-VEGF inhibitor, can reduce the production of intra-tumoral cytokines and chemokines, specifically IL-1β, IL-6 and CXCL1 [21]. Sulforaphane (SFN), an inhibitor of MIF, can also inhibit the formation of MDSCs [22]. Blocking the cytokine or chemokine receptors on MDSCs can reduce the accumulation of MDSCs. SB-265610, a specific CXCR2 antagonist, inhibits the migration of MDSCs [24]. Silibinin, a natural flavonoid from the seeds of milk thistle, can decrease the expression of CCR2 on MDSCs, which downregulates the tumor-specific homing of MDSCs [36]. In addition, inhibiting NO signaling by using NG-monomethyl-L-arginine acetate (L-NMMA), an inducible NO synthase (iNOS) inhibitor, to block MDSCs differentiation into osteoclasts was proved to be effective in MDSCs related osteolysis [18, 34].

Secondly, elimination of MDSCs is regarded as a more conventional way to target MDSCs both in basic and clinical studies. Most studies eliminated MDSCs by promoting their maturation; however, until now, the underlying mechanisms remained unclear. Activated T cells (ATC) armed with anti-CD3 × anti-Her2/neu bispecific antibodies (aATC) proved capable of eliminating MDSCs. And a Th1 cytokines-enriched (IFNγ and IL-2) microenvironment can potentiate aATC-induced suppression of MDSC [37]. A highly attenuated bacterium *Listeria monocytogenes* (Listeria^at^) can infect MDSCs and reduce the number of MDSCs in both primary tumors and blood. The remaining MDSCs are converted into an IL-12 producing immune-stimulating phenotype [38]. Cyclic di-guanylate (c-di-GMP), as a ligand for stimulator of interferon genes (STING), can interact with STING at a therapeutically low dose on the membrane of MDSC, and further convert MDSCs into an IL-12 producing immune-stimulating phenotype [39]. IL-12 can decrease the expression of nitric oxide synthase and interferon-γ mRNA to promote the maturation of MDSCs, as well as attenuate their function [40]. Adoptive cellular therapy (ACT) of reprogrammed tumor-sensitized immune cells, including activated CD25+ NKT and NK cells, as well as memory T cells, can attenuate the immune suppression of MDSC. The mechanism appears to be that activated NKT cells induce the maturation of MDSCs into DCs through NKG2D-dependent signal crosstalk, which has been verified in mice and humans successfully [41, 42]. Clinically, some methods were proven to be related to MDSC deletion. Combined injection of IL-7 and IL-15 into breast cancer lesions after radiofrequency thermal ablation (RFA) can reduce the number of MDSCs, and inhibit the growth and metastasis of breast cancer [43]. In clinical studies, surgeons found that resection of the primary tumor in metastatic breast cancer could decrease the number of MDSCs and improve patients' survival [44]. However, another performed primary tumor resection in a metastatic breast cancer mouse model, and pointed out that although resection of the primary tumor created a “window of opportunity” with decreased immune suppression, it did not affect the ultimate development of metastatic breast cancer [45]. Doxorubicin was reported to trigger apoptosis of MDSCs, leading to their elimination, *via* a mechanism related to ROS [46]. All trans retinoic acid, celecoxib, and phosphodiesterase 5 inhibitors are also reported to reduce the number of MDSCs [5, 47, 48].

Thirdly, treatments relying on reducing the levels of MDSCs' functional products might also be effective. It has been reported that 1-methyl-L-tryptophan (1-MT) can inhibit MDSC-produced IDO to suppress the inhibitory effect of MDSCs on T cells, which could help to promote the treatment of breast cancer [8].

Actually, considering the existence of MDSCs in attenuating the effect of T cells targeted immunotherapy, combination of targeting MDSCs and T cells simultaneously has been also widely explored. A *Listeria monocytogenes* (LM)-based vaccine, which expresses the tumor-associated antigen Mage-b (LM-Mb), can stimulate the anti-tumor function of CD8^+^ cells. And c-di-GMP can eliminate MDSCs by promoting maturation of MDSCs. The combined treatment of LM-Mb and c-di-GMP reduced the effect of MDSCs on CD8^+^ cells, and further augmented anti-tumor immune therapy [39]. Similarly, Curcumin can also improve the efficacy of the LM-Mb vaccine against breast cancer [35]. There was also a report on the combination of adoptive immunotherapy (AIT) using HER2-specific T cells with depletion or inhibition of MDSCs that could also augment the immune treatment [32].

## CONCLUSIONS

In breast cancer, MDSCs not only attenuate the anti-tumor immunity to promote the growth and metastasis of breast cancer, but also reduce the effect of other immune therapies. There are many methods to tackle with the impact of MDSCs, including reduction of MDSCs and inhibition of their functions. In addition, a combination of MDSC-targeted immune therapy with other types of therapies might work synergistically. Of course, there are limitations. In studies of MDSCs and breast cancer, the underlying mechanisms of MDSCs in promoting metastasis of breast cancer remain obscure. And there is little clinical evidence on the relationship between MDSCs and prognosis of breast cancer patients. The current classification of breast cancer may not be sufficiently guided for immune therapy. Patients receive the monotonous immunotherapy with varied efficiency, making it necessary to take every individual's level of MDSCs into account. Based on the facts that MSDCs have significant roles in breast cancer malignant behaviors and can be suppressed by various strategies, we do believe MDSC-targeted immunotherapy presents a broad prospect in the future.

## References

[1] Siegel RL, Miller KD, Jemal A (2016). Cancer statistics, 2016. Cancer J Clin.

[2] Gradishar WJ, Anderson BO, Balassanian R, Blair SL, Burstein HJ, Cyr A, Elias AD, Farrar WB, Forero A, Giordano SH, Goetz M, Goldstein LJ, Hudis CA, Isakoff SJ, Marcom PK, Mayer IA (2016). Invasive Breast Cancer Version 1. 2016, NCCN Clinical Practice Guidelines in Oncology. JNCCN.

[3] Filipazzi P, Valenti R, Huber V, Pilla L, Canese P, Iero M, Castelli C, Mariani L, Parmiani G, Rivoltini L (2007). Identification of a new subset of myeloid suppressor cells in peripheral blood of melanoma patients with modulation by a granulocyte-macrophage colony-stimulation factor-based antitumor vaccine. Journal of clinical oncology.

[4] Youn JI, Nagaraj S, Collazo M, Gabrilovich DI (2008). Subsets of myeloid-derived suppressor cells in tumor-bearing mice. Journal of immunology.

[5] Serafini P, Meckel K, Kelso M, Noonan K, Califano J, Koch W, Dolcetti L, Bronte V, Borrello I (2006). Phosphodiesterase-5 inhibition augments endogenous antitumor immunity by reducing myeloid-derived suppressor cell function. The Journal of experimental medicine.

[6] Zea AH, Rodriguez PC, Atkins MB, Hernandez C, Signoretti S, Zabaleta J, McDermott D, Quiceno D, Youmans A, O'Neill A, Mier J, Ochoa AC (2005). Arginase-producing myeloid suppressor cells in renal cell carcinoma patients: a mechanism of tumor evasion. Cancer research.

[7] Diaz-Montero CM, Salem ML, Nishimura MI, Garrett-Mayer E, Cole DJ, Montero AJ (2009). Increased circulating myeloid-derived suppressor cells correlate with clinical cancer stage, metastatic tumor burden, and doxorubicin-cyclophosphamide chemotherapy. CII.

[8] Yu J, Du W, Yan F, Wang Y, Li H, Cao S, Yu W, Shen C, Liu J, Ren X (2013). Myeloid-derived suppressor cells suppress antitumor immune responses through IDO expression and correlate with lymph node metastasis in patients with breast cancer. Journal of immunology.

[9] Solito S, Falisi E, Diaz-Montero CM, Doni A, Pinton L, Rosato A, Francescato S, Basso G, Zanovello P, Onicescu G, Garrett-Mayer E, Montero AJ, Bronte V, Mandruzzato S (2011). A human promyelocytic-like population is responsible for the immune suppression mediated by myeloid-derived suppressor cells. Blood.

[10] Sinha P, Clements VK, Ostrand-Rosenberg S (2005). Reduction of myeloid-derived suppressor cells and induction of M1 macrophages facilitate the rejection of established metastatic disease. Journal of immunology.

[11] Kusmartsev SA, Li Y, Chen SH (2000). Gr-1+ myeloid cells derived from tumor-bearing mice inhibit primary T cell activation induced through CD3/CD28 costimulation. Journal of immunology.

[12] Suzuki E, Kapoor V, Jassar AS, Kaiser LR, Albelda SM (2005). Gemcitabine selectively eliminates splenic Gr-1+/CD11b+ myeloid suppressor cells in tumor-bearing animals and enhances antitumor immune activity. Clin Cancer res.

[13] Duechler M, Peczek L, Zuk K, Zalesna I, Jeziorski A, Czyz M (2014). The heterogeneous immune microenvironment in breast cancer is affected by hypoxia-related genes. Journal of immunology.

[14] Waight JD, Netherby C, Hensen ML, Miller A, Hu Q, Liu S, Bogner PN, Farren MR, Lee KP, Liu K, Abrams SI (2013). Myeloid-derived suppressor cell development is regulated by a STAT/IRF-8 axis. The Journal of clinical investigation.

[15] Bergenfelz C, Larsson AM, von Stedingk K, Gruvberger-Saal S, Aaltonen K, Jansson S, Jernstrom H, Janols H, Wullt M, Bredberg A, Ryden L, Leandersson K (2015). Systemic Monocytic-MDSCs Are Generated from Monocytes and Correlate with Disease Progression in Breast Cancer Patients. PloS one.

[16] Montero AJ, Diaz-Montero CM, Deutsch YE, Hurley J, Koniaris LG, Rumboldt T, Yasir S, Jorda M, Garret-Mayer E, Avisar E, Slingerland J, Silva O, Welsh C (2012). Phase 2 study of neoadjuvant treatment with NOV-002 in combination with doxorubicin and cyclophosphamide followed by docetaxel in patients with HER-2 negative clinical stage II-IIIc breast cancer. Breast cancer research and treatment.

[17] Cao Y, Slaney CY, Bidwell BN, Parker BS, Johnstone CN, Rautela J, Eckhardt BL, Anderson RL (2014). BMP4 inhibits breast cancer metastasis by blocking myeloid-derived suppressor cell activity. Cancer research.

[18] Sawant A, Deshane J, Jules J, Lee CM, Harris BA, Feng X, Ponnazhagan S (2013). Myeloid-derived suppressor cells function as novel osteoclast progenitors enhancing bone loss in breast cancer. Cancer research.

[19] Morales JK, Kmieciak M, Knutson KL, Bear HD, Manjili MH (2010). GM-CSF is one of the main breast tumor-derived soluble factors involved in the differentiation of CD11b-Gr1- bone marrow progenitor cells into myeloid-derived suppressor cells. Breast cancer research and treatment.

[20] Oh K, Lee OY, Shon SY, Nam O, Ryu PM, Seo MW, Lee DS (2013). A mutual activation loop between breast cancer cells and myeloid-derived suppressor cells facilitates spontaneous metastasis through IL-6 trans-signaling in a murine model. BCR.

[21] Roland CL, Lynn KD, Toombs JE, Dineen SP, Udugamasooriya DG, Brekken RA (2009). Cytokine levels correlate with immune cell infiltration after anti-VEGF therapy in preclinical mouse models of breast cancer. PloS one.

[22] Simpson KD, Templeton DJ, Cross JV (2012). Macrophage migration inhibitory factor promotes tumor growth and metastasis by inducing myeloid-derived suppressor cells in the tumor microenvironment. Journal of immunology.

[23] Liu Y, Lai L, Chen Q, Song Y, Xu S, Ma F, Wang X, Wang J, Yu H, Cao X, Wang Q (2012). MicroRNA-494 is required for the accumulation and functions of tumor-expanded myeloid-derived suppressor cells *via* targeting of PTEN. Journal of immunology.

[24] Yu F, Shi Y, Wang J, Li J, Fan D, Ai W (2013). Deficiency of Kruppel-like factor KLF4 in mammary tumor cells inhibits tumor growth and pulmonary metastasis and is accompanied by compromised recruitment of myeloid-derived suppressor cells. International journal of cancer Journal international du cancer.

[25] Jaclyn Sceneay BSP (2013). Hypoxia-driven immunosuppression contributes to the pre-metastatic niche. OncoImmunology.

[26] Zhang Y, Lv D, Kim HJ, Kurt RA, Bu W, Li Y, Ma X (2013). A novel role of hematopoietic CCL5 in promoting triple-negative mammary tumor progression by regulating generation of myeloid-derived suppressor cells. Cell research.

[27] Tanaka T, Kajiwara T, Torigoe T, Okamoto Y, Sato N, Tamura Y (2015). Cancer-associated oxidoreductase ERO1-alpha drives the production of tumor-promoting myeloid-derived suppressor cells *via* oxidative protein folding.

[28] Mundy-Bosse BL, Thornton LM, Yang HC, Andersen BL, Carson WE (2011). Psychological stress is associated with altered levels of myeloid-derived suppressor cells in breast cancer patients. Cellular immunology.

[29] Vrakas CN, O'sullivan RM, Evans SE, Ingram DA, Jones CB, Phuong T, Kurt RA (2015). The Measure of DAMPs and a role for S100A8 in recruiting suppressor cells in breast cancer lung metastasis. Immunological investigations.

[30] Yu J, Wang Y, Yan F, Zhang P, Li H, Zhao H, Yan C, Yan F, Ren X (2014). Noncanonical NF-kappaB activation mediates STAT3-stimulated IDO upregulation in myeloid-derived suppressor cells in breast cancer. Journal of immunology.

[31] Lu T, Ramakrishnan R, Altiok S, Youn JI, Cheng P, Celis E, Pisarev V, Sherman S, Sporn MB, Gabrilovich D (2011). Tumor-infiltrating myeloid cells induce tumor cell resistance to cytotoxic T cells in mice. The Journal of clinical investigation.

[32] Morales JK, Kmieciak M, Graham L, Feldmesser M, Bear HD, Manjili MH (2009). Adoptive transfer of HER2/neu-specific T cells expanded with alternating gamma chain cytokines mediate tumor regression when combined with the depletion of myeloid-derived suppressor cells. CII.

[33] Oh K, Ko E, Kim HS, Park AK, Moon HG, Noh DY, Lee DS (2011). Transglutaminase 2 facilitates the distant hematogenous metastasis of breast cancer by modulating interleukin-6 in cancer cells. BCR.

[34] Sawant A, Ponnazhagan S (2013). Myeloid-derived suppressor cells as a novel target for the control of osteolytic bone disease. Oncoimmunology.

[35] Singh M, Ramos I, Asafu-Adjei D, Quispe-Tintaya W, Chandra D, Jahangir A, Zang X, Aggarwal BB, Gravekamp C (2013). Curcumin improves the therapeutic efficacy of Listeria(at)-Mage-b vaccine in correlation with improved T-cell responses in blood of a triple-negative breast cancer model 4T1. Cancer medicine.

[36] Forghani P, Khorramizadeh MR, Waller EK (2014). Silibinin inhibits accumulation of myeloid-derived suppressor cells and tumor growth of murine breast cancer. Cancer medicine.

[37] Thakur A, Schalk D, Sarkar SH, Al-Khadimi Z, Sarkar FH, Lum LG (2012). A Th1 cytokine-enriched microenvironment enhances tumor killing by activated T cells armed with bispecific antibodies and inhibits the development of myeloid-derived suppressor cells. CII.

[38] Chandra D, Jahangir A, Quispe-Tintaya W, Einstein MH, Gravekamp C (2013). Myeloid-derived suppressor cells have a central role in attenuated Listeria monocytogenes-based immunotherapy against metastatic breast cancer in young and old mice. British journal of cancer.

[39] Chandra D, Quispe-Tintaya W, Jahangir A, Asafu-Adjei D, Ramos I, Sintim HO, Zhou J, Hayakawa Y, Karaolis DK, Gravekamp C (2014). STING ligand c-di-GMP improves cancer vaccination against metastatic breast cancer. Cancer immunology research.

[40] Steding CE, Wu ST, Zhang Y, Jeng MH, Elzey BD, Kao C (2011). The role of interleukin-12 on modulating myeloid-derived suppressor cells, increasing overall survival and reducing metastasis. Immunology.

[41] Kmieciak M, Basu D, Payne KK, Toor A, Yacoub A, Wang XY, Smith L, Bear HD, Manjili MH (2011). Activated NKT cells and NK cells render T cells resistant to myeloid-derived suppressor cells and result in an effective adoptive cellular therapy against breast cancer in the FVBN202 transgenic mouse. Journal of immunology.

[42] Payne KK, Zoon CK, Wan W, Marlar K, Keim RC, Kenari MN, Kazim AL, Bear HD, Manjili MH (2013). Peripheral blood mononuclear cells of patients with breast cancer can be reprogrammed to enhance anti-HER-2/neu reactivity and overcome myeloid-derived suppressor cells. Breast cancer research and treatment.

[43] Habibi M, Kmieciak M, Graham L, Morales JK, Bear HD, Manjili MH (2009). Radiofrequency thermal ablation of breast tumors combined with intralesional administration of IL-7 and IL-15 augments anti-tumor immune responses and inhibits tumor development and metastasis. Breast cancer research and treatment.

[44] Rashid OM, Nagahashi M, Ramachandran S, Graham L, Yamada A, Spiegel S, Bear HD, Takabe K (2013). Resection of the primary tumor improves survival in metastatic breast cancer by reducing overall tumor burden. Surgery.

[45] Ghochikyan A, Davtyan A, Hovakimyan A, Davtyan H, Poghosyan A, Bagaev A, Ataullakhanov RI, Nelson EL, Agadjanyan MG (2014). Primary 4T1 tumor resection provides critical “window of opportunity” for immunotherapy. Clinical & experimental metastasis.

[46] Alizadeh D, Trad M, Hanke NT, Larmonier CB, Janikashvili N, Bonnotte B, Katsanis E, Larmonier N (2014). Doxorubicin eliminates myeloid-derived suppressor cells and enhances the efficacy of adoptive T-cell transfer in breast cancer. Cancer research.

[47] Almand B, Clark JI, Nikitina E, van Beynen J, English NR, Knight SC, Carbone DP, Gabrilovich DI (2001). Increased production of immature myeloid cells in cancer patients: A mechanism of immunosuppression in cancer. Journal of immunology.

[48] Talmadge JE, Hood KC, Zobel LC, Shafer LR, Coles M, Toth B (2007). Chemoprevention by cyclooxygenase-2 inhibition reduces immature myeloid suppressor cell expansion. International immunopharmacology.

